# Explaining progress towards Millennium Development Goal 4 for child survival in Tanzania

**DOI:** 10.7189/jogh.08.021201

**Published:** 2018-12

**Authors:** Debora Niyeha, Deogratius Malamsha, Rose Mpembeni, Debora Charwe, Saul Epimark, Khadija Malima, Akum Aveika, Tricia Aung, Elizabeth Hazel, Yvonne Tam, Rebecca Heidkamp

**Affiliations:** 1 Institute for International Programs, Johns Hopkins University. Dar es Salaam, United Republic of Tanzania; 2 National Bureau of Statistics. Dar es Salaam, United Republic of Tanzania; 3 Muhimbili University of Health and Allied Sciences. Dar es Salaam, United Republic of Tanzania; 4 Tanzania Food and Nutrition Centre. Dar es Salaam, United Republic of Tanzania; 5 Tanzania Commission for Science and Technology. United Republic of Dar es Salaam, Tanzania; 6 Department of International Health. Johns Hopkins University Bloomberg School of Public Health, Baltimore, Maryland, USA

## Abstract

**Background:**

During the Millennium Development Goal (MDG) era (1990-2015) the government in Mainland Tanzania and partners launched numerous initiatives to advance child survival including the comprehensive One Plan for Maternal Newborn and Child Health in 2008-2015 and a “sharpened” One Plan strategy in early 2014. Moving into the Sustainable Development Goal era, the government needs to learn from successes and challenges of striving towards MDG 4.

**Methods:**

We expand previous work by presenting data for the full MDG period and sub-national results. We used data from six nationally-representative household surveys conducted between 1999 and 2015 to examine trends in coverage of 22 lifesaving maternal, newborn, child health and nutrition (MNCH&N) interventions, nutritional status (stunting; wasting) and breastfeeding practice across Mainland Tanzania and sub-nationally in seven standardized geographic zones. We used the Lives Saved Tool (LiST) to model the relative contribution of included interventions which saved under 5 lives during the period from 2000-2015 compared to 1999 on a national level and within the seven zones.

**Findings:**

Child survival and nutritional status improved across Mainland Tanzania and in each of the seven zones across the 15-year period. MNCH&N intervention coverage varied widely and across zones with several key interventions declining across Mainland Tanzania or in specific geographical zones during all or part the period. According to our national LiST model, scale-up of 22 MNCH&N interventions – together with improvements in breastfeeding practice, stunting and wasting – saved 838 460 child lives nationally between 2000 and 2015.

**Conclusions:**

Mainland Tanzania has made significant progress in child survival and nutritional outcomes but progress cannot be completely explained by changes in intervention coverage alone. Further examination of the implementation and contextual factors shaping these trends is important to accelerate progress in the SDG era.

Tanzania’s transition from the Millennium Development Goals (MDG) to adoption of the Sustainable Development Goals (SDG) has prompted national discussions on how to best implement programs and measure progress towards updated targets [1]. Key to meeting SDG 3 – Good Health and Well-Being – is learning from Tanzania’s achievements and shortcomings in implementation of maternal, newborn, child health, and nutrition (MNCH&N) interventions during the MDG period.

Since 1990, Government of Tanzania launched numerous initiatives to improve maternal, newborn, and child health. Key milestones include the Integrated Management of Childhood Illness (IMCI) strategy (1996), introducing vitamin A supplementation for mothers and children (1997), developing a National Strategy on Infant and Young Child Feeding and Nutrition (2005) and National Road Map Strategic Plan to Accelerate the Reduction of Maternal and Newborn Mortality “One Plan” (2008), and as well as the introduction of new vaccines including HiB (2009), pneumococcal conjugate (2013) and rotavirus (2013) [2-4].

Over the same period there have been rapid improvements in child survival (MDG 4), yet slower progress reducing maternal mortality (MDG 5). Two sets of projections released prior to the 2015 MDG endline anticipated different outcomes in terms of meeting MDG 4. The United Nations Inter-agency Group for Child Mortality Estimation (UN-IGME) projection showed Tanzania achieving the MDG 4 target of 54 per 1000 live births by 2012, however, a projection from the Institute for Health Metrics and Evaluation suggested that the country would not meet MDG 4 [4,5]. In 2013, the Tanzanian Countdown Country Case Study Group published a policy brief and subsequent manuscript detailing the progress in newborn, child, and maternal survival [4,6]. Using data available at the time (1999-2012) they attributed Tanzania’s mixed progress towards MDG targets to differences in funding and political prioritization with interventions targeting young children receiving more attention than those targeting newborns or women. The Case Study findings informed development of a “sharpened” One Plan to accelerate gains in MNCH&N outcomes during the final two years of the MDG period [7].

In 2015-16, Tanzania conducted a Demographic and Health Survey and Malaria Indicator Survey (TDHS-MIS). These new data allow us to examine Tanzania’s progress in MNCH&N intervention coverage and health outcomes at national and sub-national levels across the full MDG period. We present findings from an analysis completed by the National Bureau of Statistics (NBS) together with representatives of other Government of Tanzania MNCH&N stakeholders contributing to the National Evaluation Platform (NEP) Tanzania.

## METHODS

We examined coverage trends of key MNCH&N interventions and nutrition outcomes based on data available in nationally-representative household surveys and used the Lives Saved Tool (LiST) (Version 5.35) to model the impact of coverage change on newborn and child mortality between 1999 and 2015. All analyses excluded Zanzibar as the NEP engages Mainland Tanzania stakeholders only.

We recalculated all MNCH&N coverage and nutrition outcome indicators from the original survey data sets using a standardized definition to ensure they are comparable across time, survey type, and sub-national units (see **Online Supplementary Document(Online Supplementary Document)** – Indicator Definition). We identified publicly-available nationally-representative MNCH&N surveys conducted between 1990 and 2016. We chose to omit data from surveys before 1999 including Tanzania Demographic and Health Surveys (TDHS) for 1991-2 and 1996 due to a reduced number of key intervention indicators, data quality concerns due to higher rates of age heaping in children and in 1991-2 small sample sizes that could not support sub-national analyses. Thus, we considered 1999 our baseline year for our analysis and used coverage and nutritional status estimates from TDHS 1999, 2005, 2010 and 2015-6. Two additional years of data for malaria-related interventions were obtained from nationally-representative HIV and Malaria Indicator Surveys (THMIS) in 2007 and 2012.

Between 2010 and 2015, the Government of Tanzania made changes to administrative boundaries at district and regional levels, which has changed the zonal classifications used by the National Bureau of Statistics to report nationally-representative household surveys. Mainland Tanzania was divided into seven zones before 2012 and eight zones after 2012. To make sub-national estimates comparable over time, we recalculated indicators from the original household survey data sets using seven zones standardized to TDHS 2005 boundaries (**Table 1**). For the survey data sets that included geocoded data for each enumeration area (TDHS 2010 & 2015-6; THMIS 2007 & 2012), we mapped them against 2002 Census shape files to identify standardized zone. For TDHS 1999, we used the regional variable in the data set to assign zones.

**Table 1:** Zonal definitions based on TDHS 2004-5 boundaries and estimated population

Zone	Regions	Estimated population (Census 2012)
Western	Tabora, Kigoma, Shinyanga	7 538 518
Northern	Arusha, Kilimanjaro, Tanga, Manyara	6 804 733
Central	Dodoma, Singida	3 454 225
Southern Highlands	Iringa, Mbeya, Rukwa	5 919 888
Lake	Kagera, Mwanza, Mara	8 713 892
Eastern	Morogoro, Pwani, Dar es Salaam	5 125 667
Southern	Lindi, Mtwara, Ruvuma	3 512 397

**LiST models.** We used LiST Version 5.35 to create retrospective models of MNCH&N coverage change from 1999-2015 in Mainland Tanzania and in each of the seven zones. LiST is a modelling software that uses coverage and known efficacy of key MNCH&N interventions to model and attribute health impact to interventions which contribute to reductions in mortality. Using LiST, we calculated the number of lives saved due to scaling-up specific interventions relative to their coverage levels during the baseline year (1999). LiST includes more than 75 MNCH&N interventions, practices and risk factors. Data sources for LiST assumptions are documented in the software and elsewhere [8]. The baseline mortality estimate for Mainland Tanzania are from UN-IGME and, we adjusted the Mainland estimates for zones using the LiST approach which uses patterns of sub-national coverage to recompute baseline mortality and cause of death distributions at the sub-national level. National and sub-national population data were obtained from the Tanzania Population and Housing Census 2002 and 2012.

LiST models changes in cause of death by starting with country-specific estimates from the World Health Organization-Maternal and Child Epidemiology Estimation group at baseline (1999) and adjusts based on the measured changes in intervention coverage.

LiST offers two approaches for modeling changes in nutritional status (prevalence of stunting and wasting) and breastfeeding practice into models. The intervention-based approach requires input of population-based coverage data for behavior change communication and supplementation programs among children 0-23 months of age as well as community-based management of acute malnutrition programs among children 6-59 months. These data were not available from population-based surveys so we adopted the alternative “direct entry” approach that tracks the prevalence of stunting, wasting and age-appropriate breastfeeding practices entered into the model without data on the potentially contributing interventions.

For our primary scenario, we used measured coverage estimates from the household surveys to model the actual trends. We then modeled alternative scenarios to help quantify the impact of stagnant or declining coverage for specific interventions (eg, scenarios if all zones had reached the same coverage as the highest performing zone). We also modeled a scenario for elimination of stunting and wasting by assuming a normal distribution across z-score categories in order to quantify the number of additional deaths due to malnutrition.

## RESULTS

### Population growth, mortality and causes of death in children under 5

According to the census, the total population of Mainland Tanzania increased by 30% between 2002 and 2012. The 0-59 month-old population increased 25% during the same period, raising the total number of children under 5 years of age who need to be reached with key preventative interventions from 5.5 million in 2002 to 7.1 million in 2012.

Child mortality in The United Republic of Tanzania was 67 per 10000 live births (95% CI = 60-74) in 2010-2015 compared to 107 in early 2000s [9]. The country did not reach the MDG 4 target of 54 deaths per 1000 live births. The under 5 mortality rate for 2005-2010 in Mainland Tanzania was 79 (95% CI = 73-84), significantly higher than 56 (95% CI = 23-67) in Zanzibar. Less progress has been made in reducing neonatal mortality over the same period. There was no difference in neonatal mortality for 2005-2015 between Mainland Tanzania (29, 95% CI = 25-33) and Zanzibar (28, 95% CI = 19-36).

**Figure 1:**
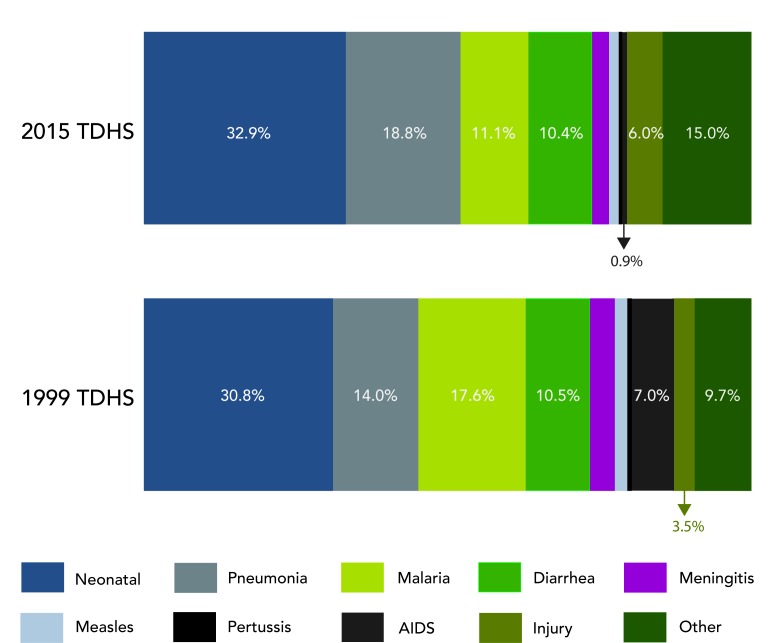
Distribution of under-five deaths in Mainland Tanzania.

**Figure 2:**
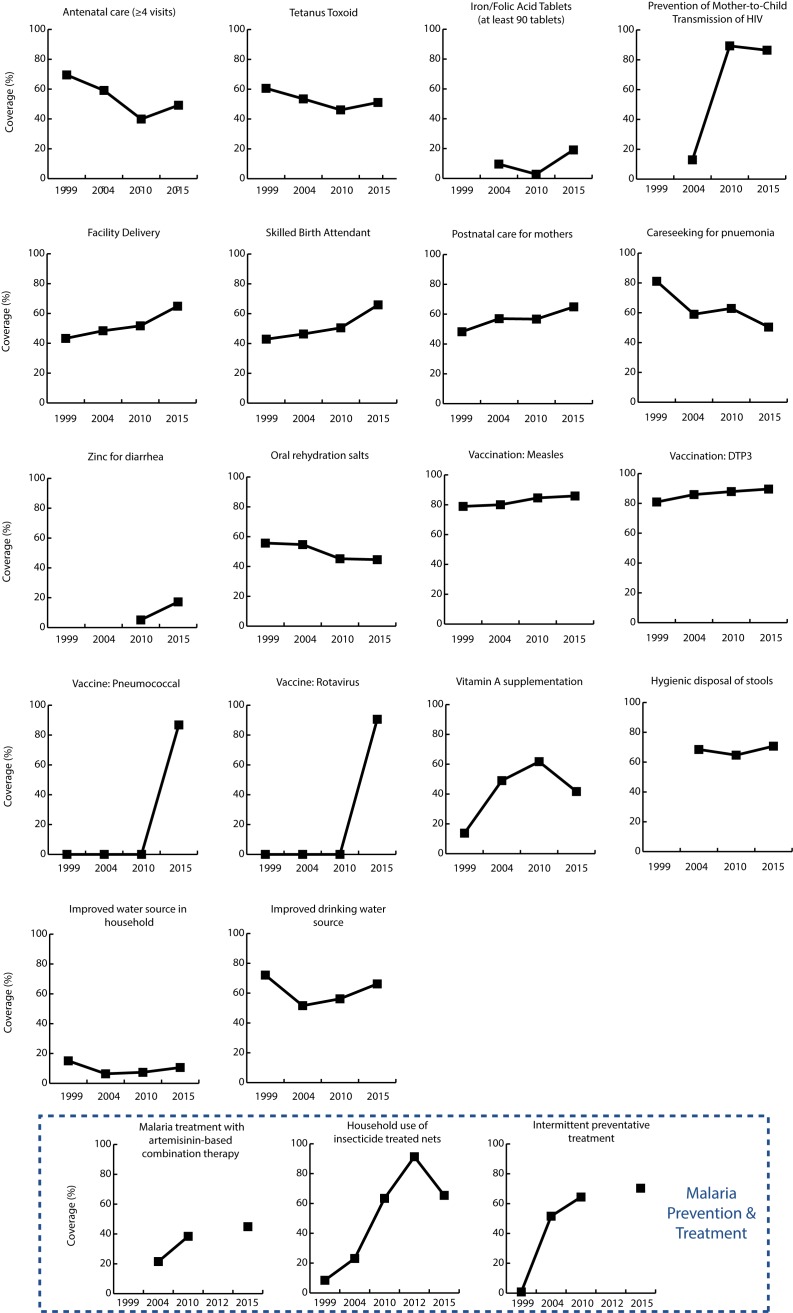
Coverage of interventions in Mainland Tanzania (2000-2015).

Causes of death modeled in LIST for children under 5 in Mainland Tanzania did not change substantively between 1999 and 2015 except for malaria and HIV/AIDS (**Figure 1**). According to the model, about one-third of deaths are attributable to neonatal causes (30.8% in 1999; 32.9% in 2015). Prematurity is responsible for one-quarter of all neonatal deaths with asphyxia and sepsis as other leading causes. Together diarrhea, pneumonia and malaria remain the leading killers of children under 5, responsible for 42.1% of under 5 deaths in 1999 and 40.3% in 2015. There is a notable decline in the proportion of deaths due to HIV-related causes, 7.0% in 1999 compared to 0.9% in 2015. This parallels to increases in PTMCT coverage (**Figure 2**) and other HIV prevention and treatment interventions.

### Changes in MNCH&N Intervention Coverage

There was variable progress in the scale up of childhood interventions at Mainland and zonal levels. According to our model, scale-up of 22 MNCH&N interventions since 1999 together with improvements in breastfeeding practice, stunting and wasting saved 838 460 child lives between 2000 and 2015.

**Figure 3:**
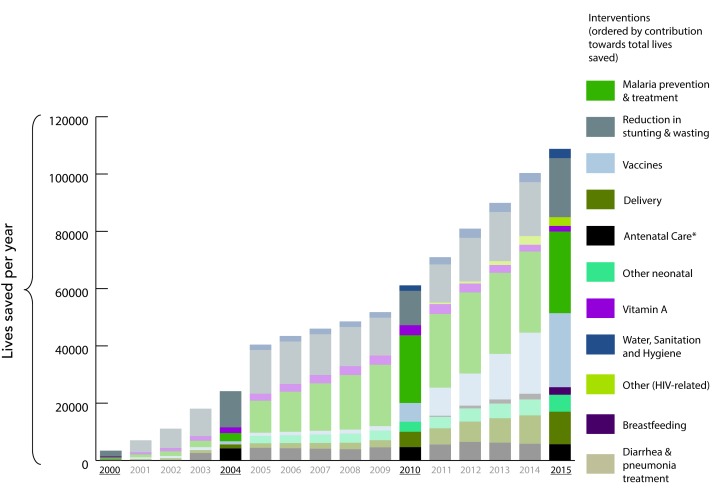
Under-five lives saved by intervention group in Mainland Tanzania (2000-2015). DHS conducted in 1999, 2004, 2010, and 2015. *Excludes intermittent preventative treatment (IPTp) which is accounted for in "Malaria prevention treatment".

**Figure 4:**
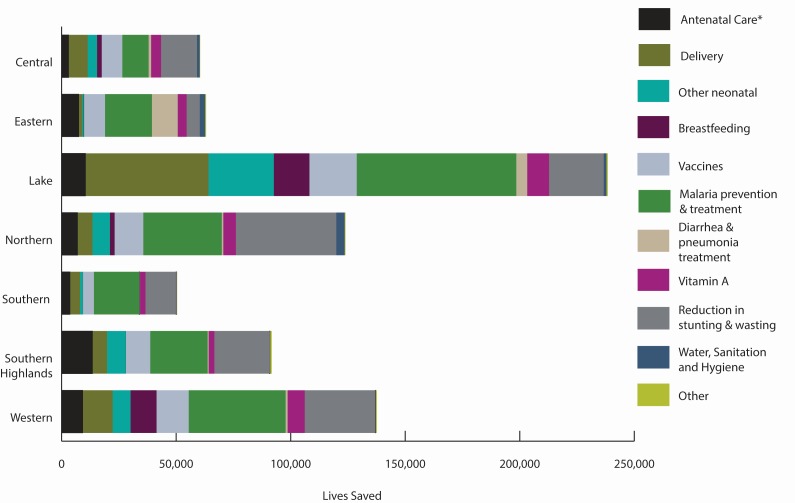
Under-five lives saved by zone in Mainland Tanzania (2000-2015).

Interventions around delivery, childhood vaccinations, and malaria prevention and treatment consistently ranked among the largest contributors to lives saved across the seven zones (**Figure 3**). Improvements in exclusive breastfeeding practices saved many additional lives in Western and Lake zones while increased coverage in interventions for management of diarrhea and pneumonia were key contributors in Eastern zone. Differences in total lives saved by zone are in part due to population size. Lake and Western zones have more than twice the total population of Central and Southern zones (**Figure 4**).

**Interventions around delivery.** There were consistent increases in coverage of interventions related to birth (facility delivery; skilled birth attendant) and postnatal care for women across Mainland Tanzania. Eastern zone achieved the highest coverage for all three delivery and postnatal interventions while Western and Lake zones had the lowest coverage of these interventions in both 1999 and 2015. Prevention of mother to child transmission (PMTCT) coverage in Mainland Tanzania scaled up rapidly from the year 2000 onwards reaching over 85% in 2015. We estimated that 61 000 child lives were saved due to scale-up of facility delivery. However, the variable progress in intervention scale-up across zones signifies a missed opportunity. If all zones had followed the same pattern as the best-performing Eastern zone, Mainland Tanzania would have saved 126 579 under 5 lives over the 15 years.

Scale-up of PMTCT services contributed to 70 000 under 5 lives saved in Mainland Tanzania over the 15-year period, the largest single contributor to lives saved after malaria-related interventions (**Table 2**). The Government of Tanzania adopted WHO Option B+ in 2012 and subsequently launched a new 3-year PMTCT strategy (2013-2015). Between 2010 and 2015, coverage of PMTCT services among HIV-infected pregnant women exceeded 85%.

**Table 2:** Under-5 (U5) lives saved by intervention relative to 1999, Mainland Tanzania

Interventions	Lives Saved 2000-2015	Intervention combination
Malaria prevention and treatment	253 261	ACT, IPTp, ITN
Changes in stunting and wasting	203 587	Stunting and wasting
U5 Nutrition intervention	45 252	BF, Vitamin A supplementation and measles treatment, iron and multiple micronutrient
Vaccines	101 803	Hib, DPT, PCV, Rota, measles
WASH	46 439	Hygienic disposal of stool, improved sanitation
Delivery interventions	61 709	Labor and delivery management, neonatal resuscitation, clean postnatal, clean birth, immediate stimulation, Antibiotic for PROM
HIV prevention interventions	78 849	PMTCT, ART, cotrimoxazole
Others	149	maternal birth order and birth intervals
Curative interventions after birth	47 411	Case management of sepsis and pneumonia, zinc for treatment of diarrhea and case management for premature babies
Total Lives Saved	838 460	

ACT – artemisinin-based combination therapy, IPTp – Intermittent preventative treatment in pregnancy, ITN – insecticide-treated bednet, WASH – Water sanitation and hygiene, DPT – diphtheria, tetanus, pertussis, PCV – pneumococcal vaccine, PROM – premature rupture of membranes, PMTCT – prevention of mother to child transmission, ART – anti-retroviral therapy

**Childhood vaccinations.** Three new vaccines have been introduced in Mainland Tanzania since 2008; by 2015, coverage of all child vaccines reached equal or greater than 80%, except for pneumococcal in Western Zone at 75% (**Figure 2**). Hib vaccine was particularly important, saving 69 000 lives – equivalent to 8% of all lives saved in Mainland Tanzania between 2000 and 2015. Diphtheria-tetanus-pertussis (DTP) and measles vaccines had a limited impact on lives saved since coverage of these vaccines was already high across all zones in 1999 (see **Online Supplementary Document(Online Supplementary Document)** – Vaccine Details).

**Figure 5:**
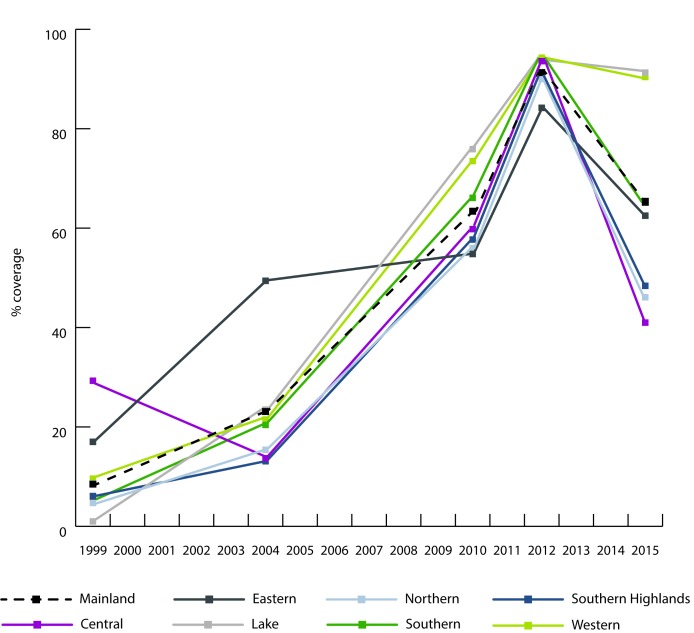
Insecticide-treated net coverage by zone in Mainland Tanzania (1999-2015).

**Malaria prevention and treatment.** There were modest gains in malaria treatment coverage across Mainland Tanzania after artemisinin-based combination therapy (ACT) was introduced in the early 2000s. Southern zone had the largest gains in treatment coverage compared to all other zones, reaching 60.5% in 2015 compared to 27.1% in 2004. In contrast, Central zone only increased ACT coverage by 7% points since 2004, reaching 21.4% in 2015. Overall an estimated 106 000 child lives were saved between 2000-2015 due to ACT scale-up (**Table 2**). Intermittent preventative treatment in pregnancy (IPTp) coverage gradually increased after it was introduced in 2000 and reached levels in 2015 comparable to coverage of four or more antenatal care visits (ANC 4+). ANC is the platform through which IPTp is delivered but the protocol only requires two visits to complete. As a result, IPTp coverage is consistently higher than ANC 4+ coverage. IPTp coverage has increased in all zones since 2004. However, the total number of child under 5 lives saved due specifically to IPTp is relatively small (**Table 2**). In contrast, insecticide-treated bednets (ITNs) were the intervention that made the single largest contribution across the 15 years with almost 145 000 under 5 lives saved. (**Table 2**). ITN coverage in Mainland Tanzania increased rapidly between 2005 and 2012 reaching 91.3% but then fell to 65.4% in 2015. Two zones, Lake and Western, were able to maintain high ITN coverage over the last five years while ITN coverage in three zones (Central, Northern, Southern Highlands) fell below 50%. (**Figure 5**).

Between 1999 and 2015, coverage of several key interventions was stagnant, while coverage of other interventions declined.

**Antenatal care.** Little progress was made in improving care during pregnancy. ANC 4+ along with several associated interventions (eg, Tetanus Toxoid, iron/folic acid) were lower in 2015 compared to 1999 across all zones. However, there was a relative increase between 2010 and 2015. ANC 4+ patterns varied by zone with Eastern having highest coverage in both 1999 (92.7%) and 2015 (73.7%) (see **Online Supplementary Document(Online Supplementary Document)** – Coverage Trends, Zone). Coverage of IFA has increased across Mainland Tanzania, particularly over the last 5 years, with most women having received at least one dose (82.0%), but proportion of pregnant women receiving at least 90 pills is extremely low at 19.1% in 2015 (**Figure 2**). Excluding PMTCT, very few additional lives were saved due to interventions during pregnancy (**Table 2**).

**Treatment of pneumonia and diarrhea.** Care seeking for pneumonia declined across all zones since 1999. In 2015 four zones had coverage at or below 50% (Central, Lake, Southern Highlands, Western) while Southern zone had the highest coverage at 73.4% (see **Online Supplementary Document(Online Supplementary Document)** – Coverage Trends, Zone). With consistently declining coverage, the intervention did not save any lives in our models (**Figure 3**). ORS coverage in Mainland Tanzania has remained stagnant; 55.4% in 1999 down to 44.6% in 2015 (**Figure 2**). The pattern at zonal level has varied slightly, with net 15 000 lives saved across the period in Eastern and Lake zones. These results must be interpreted in light of small sample sizes at zonal level (**Figure 4**).

**Vitamin A.** After steady gains between 1999 and 2010, Vitamin A coverage fell across Mainland Tanzania by 21 percentage points between 2010 and 2015. Five zones had Vitamin A coverage below 50% in 2015 with the lowest in Western zone where only 1 in 4 children under 5 received vitamin A in previous 6 months (see **Online Supplementary Document(Online Supplementary Document)** – Coverage Trends, Zone). If instead of declining Mainland Tanzania had maintained 2010 coverage levels in 2015, 1958 under 5 lives would have been saved relative to 1999. The small number is due to the prevention of measles through vaccination.

**Water sanitation and hygiene (WASH).** There has been little to no overall progress in WASH intervention coverage across Mainland Tanzania since 1999 with water-related indicators slowly moving back towards 1999 levels over the last 10 years and other WASH indicators holding fairly steady across the 15 years (**Figure 2**). This lack of meaningful improvement in coverage is reflected as missed opportunities to save lives in the LiST model.

### Contribution of stunting and wasting to mortality

**Figure 6:**
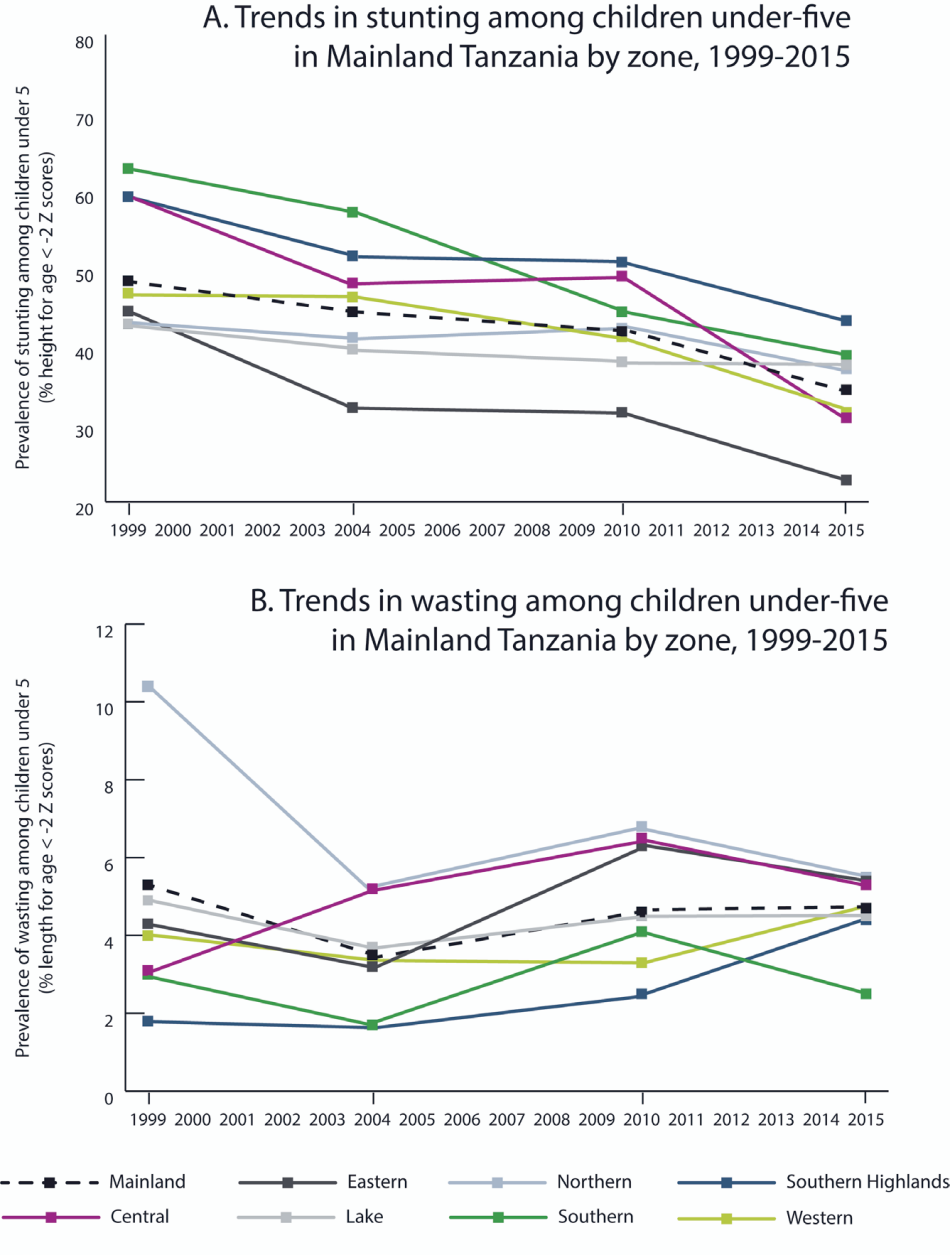
Stunting and wasting among under-five children in Mainland Tanzania (1999-2015).

The stunting burden in Mainland Tanzania has reduced from 48.4% 1999 to 34.4% in 2015. There is statistically significant variation in the prevalence of stunting at zonal level (**Figure 6**). Two zones, Central and Eastern, had nearly 50% declines in stunting and wasting relative to 1999 compared to that in Lake (12.3% decline) and in Northern (13.2% decline) zones. Eastern had the lowest stunting prevalence in 2015 at 22.8%. Southern zone had the highest stunting prevalence in 1999 (62.8%) which reduced to 38.2% in 2015. The relative stunting decline in Western zone was moderate (15.3%) but given lower stunting prevalence in 1999, it remained among the lowest across the zones in 2015 at 31.5%. Wasting remained relatively constant across the period (**Figure 6**). Northern zone had the highest burden of wasting in 1999 which reduced nearly 50% by 2015. Central and Southern Highlands zones experienced increases over the same period. Wasting is a relatively rare event and susceptible to acute seasonal fluctuation. As such these trends should be interpreted with caution.

**Figure 7:**
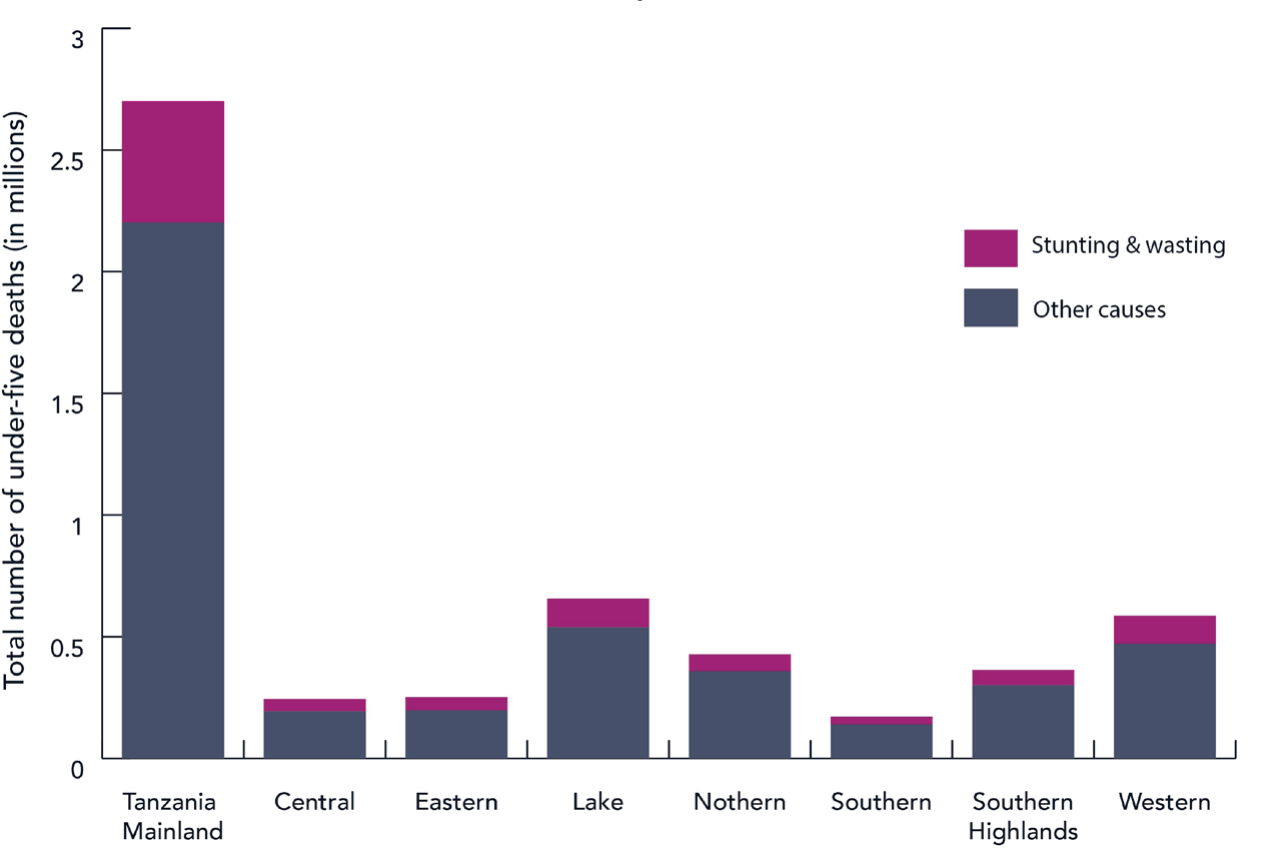
Under-five deaths due to stunting and wasting compared to other causes by zone (2000-2015).

Malnutrition is an underlying cause of child death; a malnourished child is more likely to die from infectious causes than a child who is adequately nourished [10,11]. According to our model, the reduction in stunting contributed to 12% of the lives saved in Mainland Tanzania between 2000 and 2015 (**Table 2**). Changes in wasting contributed to 12.5% of all lives saved in Mainland Tanzania between 2000 and 2015 compared to that in 1999 (**Table 2**). Despite progress in stunting reduction, a high burden of malnutrition persists in Mainland Tanzania. According to our model, about half a million of the under 5 deaths in the past 15 years were attributable to stunting or wasting (**Figure 7**).

### Changes in breastfeeding practice

Early initiation of breastfeeding is an important practice for neonatal survival and establishing exclusive breastfeeding practice. Early initiation of breastfeeding improved progressively in Mainland Tanzania from 40%, in 1999 to 63% in 2015. The rates of any breastfeeding in both 6-11 month-olds and 12-24 month-old olds have held constant over last 15 years (98.1% and 75.4% respectively in 2015). Overall these improvements in breastfeeding practice contributed to 7840 lives saved between 2000 to2015 compared to that in 1999. Small sample sizes restrict our ability to assess breastfeeding practices at zonal level.

### Comparison of measured and modeled mortality rates

Our LiST Mainland Tanzania national model predicted a mortality drop of 48 points between 1999 and 2015 compared to the 80-point drop measured by TDHS surveys in that period (see **Online Supplementary Document(Online Supplementary Document)** – LiST TDHS Mortality Comparison).

## DISCUSSION

Our analysis, completed by team of Government of Tanzania stakeholders through the National Evaluation Platform (NEP) Tanzania, examines the contributions of changes in MNCH&N interventions to estimate declines in under five mortality between 1999 and 2015. The United Republic of Tanzania has made considerable progress in reducing child mortality over the last 15 years. However, despite the optimism of certain projections, the country did not meet the MDG 4 target for child survival. Mainland Tanzania lags behind Zanzibar in reducing child mortality.

This lag can be partially explained by the variable and weak progress in scaling-up select key interventions in Mainland Tanzania. In our mainland Tanzania LiST model, changes in coverage of 22 MNCH&N interventions together with changes in the prevalence of stunting, wasting and breastfeeding practices explained about 60% of the 15-year mortality decline. Other factors not examined in our analysis are important contributors to the mortality decline.

The Tanzania Countdown Case study team provided a comprehensive analysis of trends in mortality, MNCH intervention coverage and equity in Mainland Tanzania through 2010 (2012 for HIV and malaria interventions) [4]. Their analysis directly informed development of the Sharpened One Plan for MNCH in 2013, which aimed to accelerate progress in the final two years of the MDG period. Noting the slow improvement in neonatal and maternal survival and the need to sustain child mortality declines, the sharpened plan called for focused efforts in three program areas: improved care around delivery for mother and newborns, access to family planning for adolescents and universal coverage in prevention and treatment of childhood illnesses including malaria, pneumonia, diarrhea, HIV & malnutrition. The revised plan also called for concentrated efforts for maternal interventions in two consistently underperforming geographical zones, Lake and Western. We center our discussion on trends over the last 5 years of the period (2010-2015), where the more recent data from the TDHS 2015 adds new insights to the Countdown team’s analysis and allows for a high-level assessment of overall Sharpened One Plan impact.

Based on the 5-year coverage changes for pregnancy, delivery and child health interventions compared to operational targets set out in the Sharpened One Plan, Mainland Tanzania failed to achieve its primary goals of accelerating improvements in both the three program areas and in the poorly performing regions of Lake and Western. The largest absolute increase in coverage across Mainland Tanzania in the last five years of the MDG period was for ANC4+ visits. However, this followed dramatic declines in ANC4+ coverage over the previous 10 years so that by 2015 only 2 out of 3 pregnant women attended at least four visits compared to an operational target of 90%. Improvements were not consistent across zones with Lake and Western performing much worse than other zones in terms of both the 5-year rate changes in intervention coverage and coverage of interventions in 2015. ANC is the delivery platform for several interventions important for maternal and neonatal survival including IPTp, Tetanus Toxoid and IFA. In 2015, coverage of both TT and IFA interventions was below that of ANC4+, suggesting that even if overall women are attending more ANC visits, the quality of that service remains poor as key components are not reaching women.

Both 5- and 15-year trends in coverage of delivery platforms surrounding delivery (ie, facility delivery, skilled birth attendance, postnatal care for mothers), were consistently positive across zones but they varied in both the achieved coverage and the rate of change. However, unlike ANC, direct measures of coverage for the sub-interventions (eg, neonatal resuscitation, antibiotics for PROM, etc.) were not available so coverage change was modeled based on standardized LiST assumptions. Several zones including Eastern and Southern reached coverage over 80% for all three delivery platforms, providing evidence that the Sharpened One Plan operational target for Skilled Birth Attendants was reasonable and achievable within the broader context of the Tanzanian health system. However, the prioritized zones of Lake and Western did not perform well in terms of improving coverage of the three delivery-related interventions. Lake zone remained the poorest performer for all three interventions in both absolute coverage and rate of change over the last 5 years. In 2015, coverage of the three delivery-related interviews was also low in Western Zone; however, coverage of these interventions improved slightly over the past five years.

Weak progress in childhood interventions points to insufficient implementation of the One Plan over the last 5 years. The plan laid out a target of 90% of children seeking appropriate care for illness. Although malaria-related interventions saved a relatively large number of child lives since 1999, coverage of malaria prevention (ITN) and treatment interventions remain low across Mainland Tanzania. Coverage of malaria-related interventions over the past five years vary greatly across zones; there were net decreases in both ITNs and ACT coverage in 3 zones. In contrast to the other program areas, Lake and Western demonstrated large 5-year gains in ITN coverage and achieved the highest ITN coverage in 2015 (above 90%, the plan’s target for children sleeping under a net). Western zone was also the highest performer for increases in ACT treatment, but still only half of children who needed intervention received it. Despite 15-year net increases, vitamin A coverage fell dramatically across all zones over the last five years. Pneumonia careseeking started high at the 1999 baseline and fell steadily across the period. Vaccination coverage, including a number of new vaccines introduced in the last five years is relatively high across zones (except Western) but did not quite achieve the target of 90% DPT3 and measles coverage in 90% of zones. PMTCT coverage was also successfully scaled above the 77% target for HIV-infected pregnant women and has remained high.

Others have noted the importance of Tanzania’s high population growth rate in explaining some of the stagnation or declines in coverage [4]. Additional inputs (eg, available supplies and staffing) are required to maintain the same coverage across a larger population. Lake zone – and to a lesser extent Western zone – had high numbers of lives saved even with relatively low coverage of interventions due to large populations in these zones. It is also important to note that interventions that already have high coverage at the start of the period (eg, vaccines) did not contribute many additional lives saved in our model. However, those interventions that had meaningful declines in coverage were captured by the models in terms of both having no impact (ie, lives saved) and in alternate scenarios.

In the Countdown Case Study analysis, less attention was paid to the contribution of changes in nutritional status to mortality reduction. Declines in stunting and wasting explain about one-quarter of the under 5 lives saved our model. Our use of the “direct entry” method for stunting, wasting and breastfeeding practices captures these intervention impacts in a non-specific way. Global estimates suggest that undernutrition contributes to nearly half of the child mortality burden in contexts like Tanzania with a high burden poverty and infectious illness [11]. The large number of deaths attributable to the persistent burden of malnutrition reinforces the importance of continued declines in stunting and wasting for advancing child survival. Coverage data on several important nutrition interventions including promotion of appropriate breastfeeding and complementary feeding and treatment of acute malnutrition were not available. As with child mortality, the changes in these nutrition status and practice outcomes are not likely explained by interventions effects alone. However, we lack the data needed to tease out this relationship.

The analysis used data disaggregated at zonal level as defined by Reproductive and Child Health Section of the Ministry of Health, Community Development, Gender, Elderly and Children (MoHCDGEC). The zone is not an official administrative unit in Tanzania and resource allocation is not tied to zonal entities. If sample sizes had allowed, it would have been more useful to complete the analysis at regional or district levels. However, the MoHCDGEC uses zones in strategic planning as evidenced by prioritization of Western and Lake zones in the Sharpened One Plan. Reporting by zone is useful to these stakeholders. Sub-national models required certain baseline assumptions available only at national (eg, cause of death) to be applied for sub-national regions.

The lack of quality coverage data on specific maternal and neonatal interventions is also a limitation for our analysis as we had to apply global assumptions about coverage that may not reflect actual availability of services in Tanzania. LiST does not account for the statistical uncertainty around the model inputs and outputs. With our dependence on household survey data sources that are collected every five years, we cannot draw conclusions specific to the Sharpened One Plan period. Annual utilization data from the DHIS-2 could provide a clearer picture of whether utilization increased during the period which the plan took effect but use of these data are constrained by quality issues and lack of clear denominators for coverage. The ability to apply methods for sub-national zonal analysis based upon uniform definitions from household surveys conducted cross-sectionally over time despite changing boundaries offers a distinct advantage compared to prior analyses based upon routine data sources.

### CONCLUSION

Our models clearly show that changes in MNCH intervention coverage and nutritional status are not the only drivers of mortality declines in Mainland Tanzania. If anything, under five mortality appears to have declined despite generally weak health system performance. Our alterative scenarios, particularly for malaria interventions and malnutrition, demonstrate that there is still significant potential to accelerate declines in neonatal and child mortality through investments in intervention scale-up in the areas identified under the Sharpened One Plan. Higher performing zones, including Eastern zone, demonstrate that targets for most interventions are feasible. However as the Government of Tanzania looks ahead to the SDG era and continues to align global SDG targets with national accountability initiatives including Tanzania Development Vision 2025, National Five Year Development Plan (2016/17-2020/21), and the National Road Map Strategic Plan to Improve Reproductive, Maternal, Newborn, Child, and Adolescent Health in Tanzania “One Plan II” (2016-2021), there is a critical need to dig deeper into questions of what is holding back intervention scale-up from a sub-national or zonal perspective [12]. The NEP has built capacity among key MNCH&N stakeholders in Mainland Tanzania to carry out this important process and the analyses presented here are the first step to more efficiently prioritize and advance this timely agenda.
