# Supplementary material for: Explaining progress towards Millennium Development Goal 4 for child survival in Tanzania
Source: J Glob Health. 2018 Nov 29;8(2):021201. doi: 10.7189/jogh.08.021201 (PMC6319734; doi:10.7189/jogh.08.021201)

Online Supplementary Document

Niyeha et al. Explaining progress towards Millennium Development Goal 4 for child survival in Tanzania.

J Glob Health 2018;8:021201

Appendix S1. Indicator Definition

Intervention	Definition
Antenatal care (4+)	Numerator: Number of women ages 15–49 who were attended at least four times during pregnancy in the 3 years preceding the survey; Denominator: Total number of women ages 15–49 with a live birth in the 3 years preceding the survey, most recent birth only.
Neonatal tetanus protection	Numerator: Number of mothers who received two doses of tetanus toxoid vaccine during the most recent pregancy; Denominator: Total number of women ages 15-49 with a live birth in the 3 years prior to the survey, most recent birth only. (Note: LiST projection will use the PAB UNICEF values instead of 2TT)
Intermittent preventative treatment in pregnancy (for malaria)	Numerator: Number of women ages 15–49 at risk for malaria who received ANY sulfadoxine-pyrimethamine (Fansidar™) to prevent malaria during their last pregnancy that led to a live birth in the previous 3 years; Denominator: Total number of women ages 15–49 with a live birth in the 3 years preceding the survey, most recent birth only.
Iron folate supplementation	Numerator: Number of women taking any iron-folate supplement during the most recent pregnancy that resulted in a live birth; Denominator: Total number of women with a live birth in the previous 3 years, most recent birth only
Skilled birth attendance (SBA)	Numerator: Number of women ages 15–49 with a live birth in the 3 years prior to the survey who were attended during delivery by skilled health personnel (doctors, clinical officers, nurses, midwives, and MCH aides); Denominator: Total number of women ages 15–49 with a live birth in the 3 years preceding the survey, most recent birth only
Facility delivery (clinic and hospital)	Numerator: Number of children born in an institution in the previous 3 years; Denominator: Total number of births in the previous 3 years, most recent birth only.
Early initiation of breastfeeding	Numerator: Number of women with a live birth in the 3 years prior to the survey who put the newborn infant to the breast within 1 hour of birth; Denominator: Total number of women with a live birth in the 3 years prior to the surveyed, most recent birth only.

Postnatal care for mothers	Numerator: Number of women ages 15–49 who received postnatal care within two days of childbirth (if delivered outside facility) OR all women who delivered in facility; Denominator: Total number of women ages 15–49 with a last live birth in the 3 years prior to the survey, most recent birth only.
Partial breastfeeding (<1m infant age)	Numerator: number of children (0-1m) receiving breastmilk plus complementary foods and/or milk-based liquids (plus medication, vaccines, and vitamins); Denominator: Total children 0-1m of age
Partial breastfeeding (1-5m infant age)	Numerator: number of children (1-5m) receiving breastmilk plus complementary foods and/or milk-based liquids (plus medication, vaccines, and vitamins); Denominator: Total children 1-5m of age
Predominant breastfeeding (<1m infant age)	Numerator: number of children (0-1m) fed breastmilk plus water and/or other non-milk liquids such as juices (plus medication, vaccines, and vitamins), Denominator: Total children 0-1 months of age
Predominant breastfeeding (1-5m infant age)	Numerator: Number of children (1-5m) fed breastmilk plus water and/or other non-milk liquids such as juices (plus medication, vaccines, and vitamins), Denominator: Total children 1-5 months of age
Any breastfeeding (6-11m infant age)	Numerator: Number of children 6-11 months receiving any breastmilk; Denominator: Total number of children 6-11 months
Any breastfeeding (12-23m infant age)	Numerator: Number of children 12-23 months receiving any breastmilk; Denominator: Total number of children 12-23 months
Exclusive breastfeeding (<1m infant age)	Numerator: Number of children 0-1 months receiving only breastmilk for food (plus medication, vaccines, and vitamins).; Denominator: Total number of children 0-1 months
Exclusive breastfeeding (0-5m infant age)	Numerator: Number of infants ages 0–5 months who are exclusively breastfed; Denominator: Total number of infants ages 0–5 months surveyed
Exclusive breastfeeding (1-5m infant age)	Numerator: Number of children 1-5 months receiving only breastmilk for food (plus medication, vaccines, and vitamins).; Denominator: Total number of children 1-5 months
DPT3/Penta3 immunization	Numerator: Number of children ages 12–23 months receiving three doses of diphtheria/ pertussis/tetanus vaccine; Denominator: Total number of children ages 12–23 months surveyed. Note Pentavalent for the 2010 DHS survey (HiB included)
Rotavirus vaccination	Numerator: Number of children ages 12–23 months receiving two doses of rotavirus vaccine; Denominator: Total number of children ages 12–23 months surveyed.
Pneumococcal conjugate vaccine	Numerator: Number of children ages 12–23 months receiving three doses of pneumococcal conjugate vaccine; Denominator: Total number of children ages 12–23 months surveyed.
Measles immunization	Numerator: Number of children ages 12–23 months who are immunized against measles; Denominator: Total number of children ages 12–23 months surveyed.

Vitamin A supplementation (DHS/MICS)	Numerator: Number of children ages 6–59 months who received at least one dose of vitamin A in the previous 6 months; Denominator: Total number of children ages 6-59 months.
Zinc treatment of diarrhea	Numerator: Number of children ages 0–59 months with diarrhea in the two weeks prior to the survey receiving any treatment containing zinc; Denominator: Total number of children ages 0–59 months with diarrhea in the two weeks prior to the survey
ORS - oral rehydration solution	Numerator: Number of children ages 0–59 months with diarrhea in the two weeks prior to the survey receiving oral rehydration therapy (oral rehydration salts packet, or increased fluids); Denominator: Total number of children ages 0–59 months with diarrhea in the two weeks prior to the survey.
Malaria treatment (Any antimalarials)	Numerator: Number of children ages 0–59 months who had a fever in the two weeks prior to the survey who received any antimalarial regardless of symptom onset timing; Denominator: Total number of children ages 0–59 months who had a fever in the two weeks
Malaria treatment - Artemesinin compounds	Numerator: Number of children ages 0–59 months who had a fever in the two weeks prior to the survey who received ACT regardless of symptom onset timing; Denominator: Total number of children ages 0–59 months who had a fever in the two weeks
Care seeking for pneumonia	Numerator: Number of children ages 0–59 months with symptoms of pneumonia (cough + rapid breathing) in the two weeks prior to the survey who were taken to an appropriate health provider; Denominator: Total number of children ages 0–59 months with symptoms of pneumonia (cough + rapid breathing - no question on "problem in the chest" in the 2004 DHS) in the two weeks prior to the survey. NOTE: for the 2015 they recode h32z variable was not included, defined as hospitals (public and private), health centers (public and private), public dispensary (public and private), clinic (public and private), CHW, ADDO, NGO, Religious/voluntary medical"
Water connection in the home	Numerator: Number of households with a household connection, including water piped into the home or yard; Denominator: Total number of households.
Hygienic disposal of children's stools	Numerator: Number of children's stools that are disposed of safely and contained (use toilet, stools discarded into toilet or buried); Denominator: Total number of children age 0-3 years
Improved water source	Numerator: Number of household using improved drinking water sources [piped water, covered well, borehole, all springs (2004 DHS does not specify protected or unprotected springs), rainwater]; Denominator: Total number of households

Household ITN ownership	Numerator: Number of households owning at least 1 ITN (LLITN or treated in the previous 12m); Denominator: Total number of households. In 1999 DHS, only asked about Treated bed nets (previous 6 month) only ask whether treated if a child slept under a bed net. Did not exclude missing so can use DHS recode variables

Appendix S2. Number of Children Under-Five Estimated by Lives Saved Tool

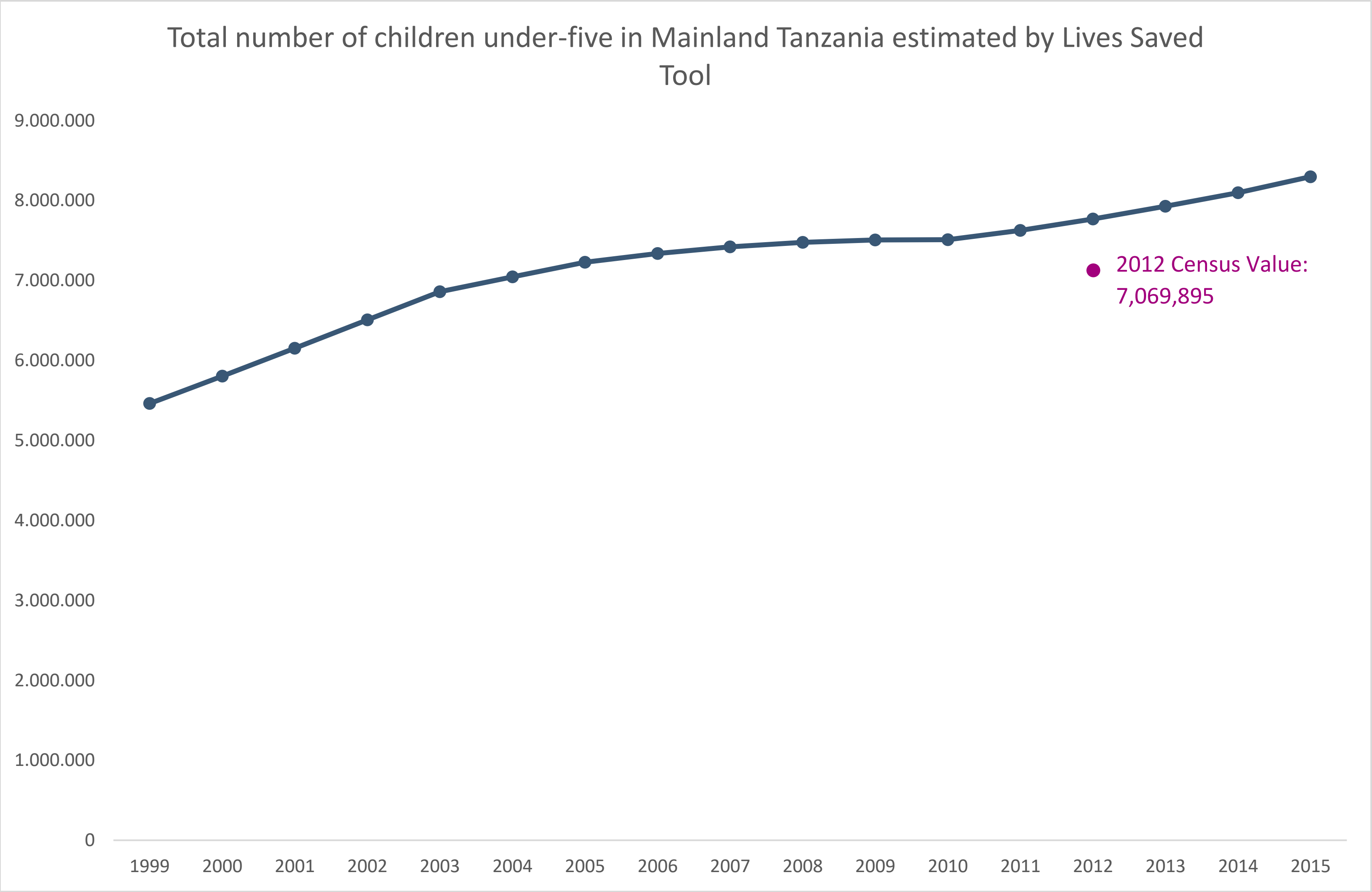

Appendix S3. Lives Saved by Vaccine in Mainland Tanzania

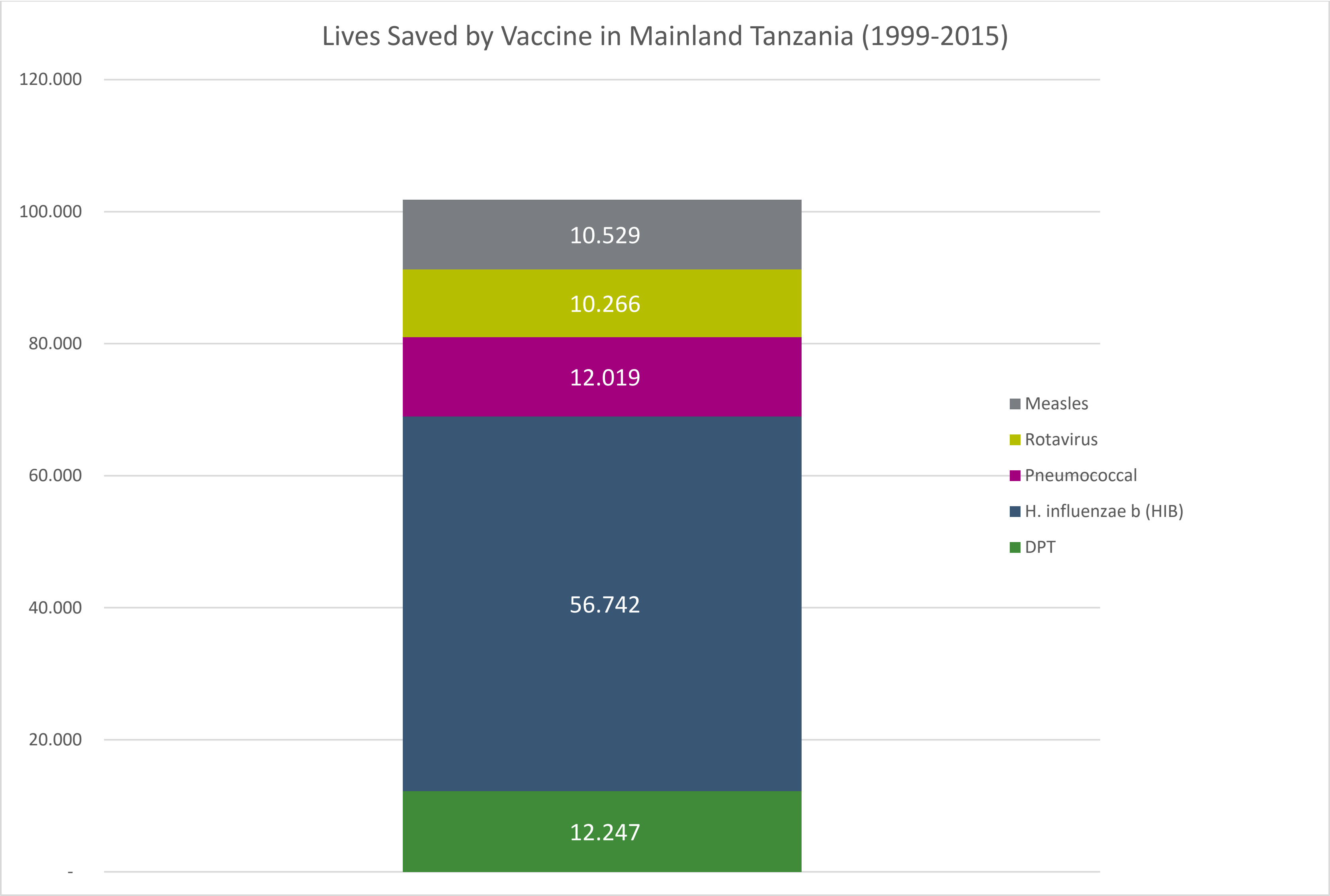

Lives Saved by Vaccine in Mainland Tanzania (1999-2015)
120.000
100.000
80.000
60.000
40.000
20.000
-
10.529
10.266
12.019
56.742
12.247
Measles
Rotavirus
Pneumococcal
H. influenzae b (HIB)
DPT

Appendix S4. Lives Saved by Vaccine by Zone in Mainland Tanzania

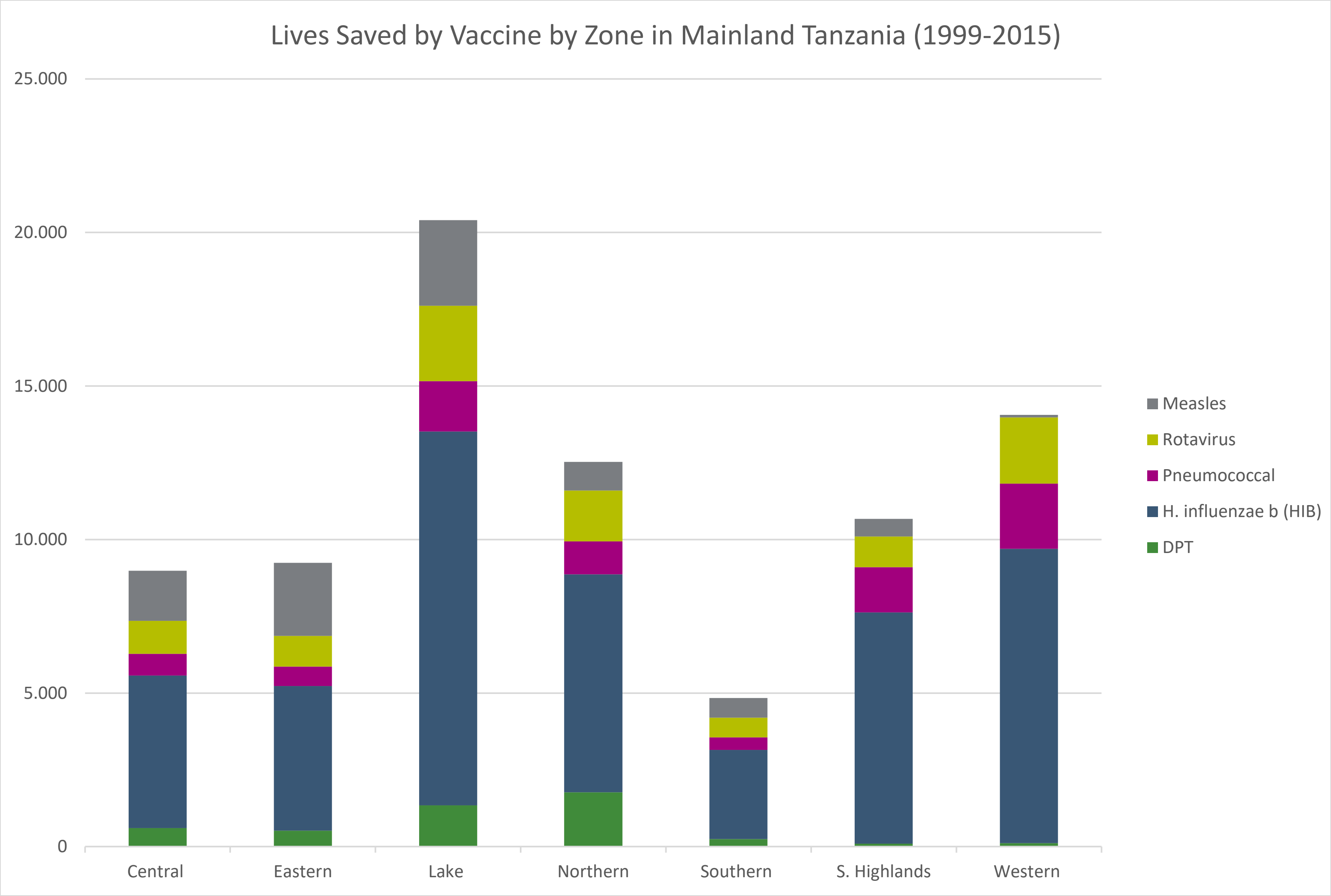

Appendix S5. Coverage of interventions during ANC by zone

ANC 4+	1999			2004			2010			2015		
	%	95% CI		%	95% CI		%	95% CI		%	95% CI	
Mainland	69.5	64.4	74.6	59.1	56.6	61.6	39.7	37.5	41.9	65.9	63.7	68.1
Central	64.5	53.1	75.9	65.8	59.1	72.5	37.5	30.4	44.6	55.2	48.5	61.9
Eastern	92.7	89.4	96.0	80.0	73.3	86.7	59.6	54.1	65.1	73.7	70.0	77.4
Lake	61.8	55.5	68.1	58.6	53.3	63.9	40.8	35.7	45.9	43.0	38.1	47.9
Northern	70.3	50.1	90.5	61.4	56.7	66.1	47.6	43.5	51.7	55.0	50.3	59.7
Southern	75.8	69.1	82.5	60.4	54.1	66.7	39.2	31.9	46.5	49.4	43.5	55.3
Southern Highlands	81.8	72.0	91.6	60.0	53.7	66.3	26.7	20.8	32.6	43.0	37.3	48.7
Western	49.4	43.9	54.9	43.2	38.1	48.3	31.8	27.7	35.9	36.2	31.5	40.9
IPTp	1999			2004			2010			2015		
	%	95% CI		%	95% CI		%	95% CI		%	95% CI	
Mainland	0.8	0.2	1.4	51.6	48.9	54.3	64.4	61.9	66.9	70.3	68.3	72.3
Central	1.0	-0.8	2.8	51.5	43.1	59.9	58.9	51.6	66.2	80.4	75.5	85.3
Eastern	1.0	-0.2	2.2	51.7	44.6	58.8	69.7	62.3	77.1	80.8	76.9	84.7
Lake	1.2	-0.4	2.8	59.7	54.0	65.4	65.8	59.7	71.9	62.5	57.4	67.6
Northern	0.5	-0.3	1.3	53.2	47.1	59.3	75.8	70.5	81.1	78.1	73.8	82.4
Southern	0.8	-0.8	2.4	64.4	58.3	70.5	71.8	65.5	78.1	78.5	72.4	84.6
Southern Highlands	0.8	-0.4	2.0	33.8	26.7	40.9	60.4	53.0	67.8	67.4	61.9	72.9
Western	0.0	0.0	0.0	50.0	44.9	55.1	55.6	51.1	60.1	61.5	56.4	66.6
Tetanus Toxoid	1999			2004			2010			2015		
	%	95% CI		%	95% CI		%	95% CI		%	95% CI	
Mainland	60.6	55.1	66.1	53.5	51.1	55.9	46.1	43.7	48.5	51.1	48.9	53.3
Central	57.8	42.9	72.7	47.2	40.3	54.1	35.7	29.4	42.0	54.8	48.1	61.5
Eastern	76.3	69.0	83.6	68.1	62.4	73.8	58.0	51.3	64.7	68.4	62.9	73.9
Lake	61.1	53.1	69.1	59.0	54.3	63.7	32.9	28.0	37.8	41.1	36.8	45.4

Northern	58.7	38.5	78.9	57.5	52.2	62.8	62.9	57.4	68.4	58.0	51.9	64.1
Southern	57.2	50.1	64.3	57.1	50.6	63.6	48.0	42.3	53.7	47.1	40.2	54.0
Southern Highlands	65.0	52.7	77.3	52.7	48.2	57.2	43.8	38.5	49.1	57.1	52.2	62.0
Western	48.8	38.0	59.6	39.9	35.6	44.2	46.8	41.3	52.3	41.3	36.6	46.0
Any IFA	1999			2004			2010			2015		
	%	95% CI		%	95% CI		%	95% CI		%	95% CI	
Mainland	46.8	41.3	52.3	61.4	59.0	63.8	57.0	54.3	59.7	82.0	80.4	83.6
Central	43.2	25.2	61.2	66.9	59.5	74.3	73.7	67.2	80.2	92.8	89.9	95.7
Eastern	53.0	43.4	62.6	55.6	48.3	62.9	66.5	61.2	71.8	91.2	88.7	93.7
Lake	45.3	34.7	55.9	59.2	54.1	64.3	49.6	42.3	56.9	77.6	73.7	81.5
Northern	36.6	21.5	51.7	58.1	52.4	63.8	57.4	52.1	62.7	85.0	82.3	87.7
Southern	48.4	36.2	60.6	81.1	74.6	87.6	69.8	63.9	75.7	84.7	79.0	90.4
Southern Highlands	64.5	51.2	77.8	61.7	56.0	67.4	51.1	42.7	59.5	82.4	79.5	85.3
Western	42.8	29.1	56.5	58.9	53.8	64.0	49.5	43.4	55.6	73.5	68.8	78.2
IFA (at least 90 tablets)	1999			2004			2010			2015		
	%	95% CI		%	95% CI		%	95% CI		%	95% CI	
Mainland	N/A	N/A	N/A	9.6	8.0	11.2	2.8	2.2	3.4	19.1	17.7	20.5
Central	N/A	N/A	N/A	8.4	5.1	11.7	6.1	3.4	8.8	23.6	18.3	28.9
Eastern	N/A	N/A	N/A	12.3	7.4	17.2	6.5	3.6	9.4	24.3	20.2	28.4
Lake	N/A	N/A	N/A	2	1.0	3.0	0.8	0.0	1.6	14.1	11.4	16.8
Northern	N/A	N/A	N/A	2.1	0.7	3.5	1.2	0.0	2.4	19.3	15.8	22.8
Southern	N/A	N/A	N/A	39.2	30.0	48.4	5.9	3.2	8.6	24.9	19.6	30.2
Southern Highlands	N/A	N/A	N/A	12.5	8.8	16.2	2.2	0.4	4.0	24.9	20.2	29.6
Western	N/A	N/A	N/A	7.3	4.9	9.7	1.1	0.3	1.9	13.7	10.8	16.6
**Interventions around delivery**												
Facility Delivery	1999			2004			2010			2015		
	%	95% CI		%	95% CI		%	95% CI		%	95% CI	
Mainland	43.3	37.8	48.8	48.4	45.1	51.7	51.7	48.6	54.8	64.9	62.2	67.6
Central	31.0	16.5	45.5	37.2	27.8	46.6	46.0	34.6	57.4	70.5	62.7	78.3

Eastern	73.5	66.6	80.4	69.5	58.7	80.3	74.9	66.7	83.1	87.5	82.8	92.2
Lake	27.7	19.1	36.3	41.8	34.7	48.9	45.2	39.1	51.3	51.1	45.2	57.0
Northern	48.6	31.5	65.7	46.8	38.0	55.6	54.0	45.0	63.0	63.0	54.4	71.6
Southern	53.1	37.2	69.0	56.5	46.9	66.1	69.9	63.4	76.4	84.1	77.2	91.0
Southern Highlands	46.6	34.8	58.4	46.0	38.0	54.0	51.7	44.3	59.1	72.4	64.6	80.2
Western	32.5	20.9	44.1	47.6	40.5	54.7	38.3	30.9	45.7	52.0	46.1	57.9

Skilled Birth Attendant	1999			2004			2010			2015		
	%	95% CI		%	95% CI		%	95% CI		%	95% CI	
Mainland	42.9	37.4	48.4	46.4	43.1	49.7	50.5	47.2	53.8	65.9	63.0	68.8
Central	30.3	17.6	43.0	39.8	30.0	49.6	46.3	35.5	57.1	71.2	63.4	79.0
Eastern	73.2	65.2	81.2	68.1	57.1	79.1	76.0	67.2	84.8	88.9	84.2	93.6
Lake	27.7	20.3	35.1	40.1	33.0	47.2	43.2	36.7	49.7	52.1	46.0	58.2
Northern	50.2	33.9	66.5	49.0	40.4	57.6	52.5	43.5	61.5	64.0	55.2	72.8
Southern	53.5	37.4	69.6	56.0	46.4	65.6	68.6	61.7	75.5	84.2	77.3	91.1
Southern Highlands	46.2	34.8	57.6	43.7	36.3	51.1	50.0	42.0	58.0	72.4	64.6	80.2
Western	30.6	18.4	42.8	41.9	35.8	48.0	38.2	30.2	46.2	53.5	47.6	59.4

Postnatal care for mothers	1999			2004			2010			2015		
	%	95% CI		%	95% CI		%	95% CI		%	95% CI	
Mainland	48.2	42.5	53.9	57.0	53.7	60.3	56.7	53.8	59.6	64.9	62.2	67.6
Central	38.2	23.3	53.1	42.7	33.1	52.3	50.2	39.8	60.6	70.5	62.7	78.3
Eastern	77.8	71.3	84.3	78.3	70.1	86.5	79.5	72.8	86.2	87.5	82.8	92.2
Lake	31.7	22.9	40.5	47.8	40.5	55.1	47.5	41.8	53.2	51.1	45.2	57.0
Northern	52.3	36.2	68.4	54.9	46.7	63.1	60.1	51.9	68.3	63.0	54.4	71.6
Southern	60.6	43.7	77.5	72.5	65.2	79.8	78.7	73.6	83.8	84.1	77.2	91.0
Southern Highlands	52.3	40.7	63.9	54.6	46.8	62.4	58.3	50.7	65.9	72.4	64.6	80.2
Western	37.6	24.9	50.3	57.4	50.3	64.5	43.1	35.1	51.1	52.0	46.1	57.9

Vitamin A & childhood vaccines

Vitamin A in past 6 months	1999			2004			2010			2015		
	%	95% CI		%	95% CI		%	95% CI		%	95% CI	

Mainland	13.8	11.3	16.3	49.0	45.9	52.1	61.7	59.5	63.9	41.7	39.5	43.9
Central	11.9	4.8	19.0	66.7	59.4	74.0	64.2	58.5	69.9	45.4	39.1	51.7
Eastern	27.7	20.4	35.0	55.3	49.0	61.6	70.7	66.2	75.2	52.5	47.2	57.8
Lake	10.4	8.0	12.8	45.0	40.3	49.7	77.6	73.7	81.5	44.9	41.0	48.8
Northern	13.4	7.5	19.3	49.1	43.0	55.2	63.1	57.2	69.0	44.7	39.4	50.0
Southern	11.0	5.7	16.3	43.5	37.6	49.4	73.2	69.3	77.1	50.9	44.4	57.4
Southern Highlands	21.8	11.0	32.6	34.8	25.4	44.2	67.1	60.0	74.2	40.0	33.7	46.3
Western	5.8	3.3	8.3	54.7	47.3	62.1	30.4	24.9	35.9	26.8	21.5	32.1
Rotavirus vaccine (12-23m)	1999			2004			2010			2015		
	%	95% CI		%	95% CI		%	95% CI		%	95% CI	
Mainland	N/A	N/A	N/A	N/A	N/A	N/A	N/A	N/A	N/A	90.6	88.2	93.0
Central	N/A	N/A	N/A	N/A	N/A	N/A	N/A	N/A	N/A	98.1	95.2	101.0
Eastern	N/A	N/A	N/A	N/A	N/A	N/A	N/A	N/A	N/A	96.8	94.4	99.2
Lake	N/A	N/A	N/A	N/A	N/A	N/A	N/A	N/A	N/A	88.9	84.0	93.8
Northern	N/A	N/A	N/A	N/A	N/A	N/A	N/A	N/A	N/A	97.1	94.9	99.3
Southern	N/A	N/A	N/A	N/A	N/A	N/A	N/A	N/A	N/A	95.0	90.7	99.3
Southern Highlands	N/A	N/A	N/A	N/A	N/A	N/A	N/A	N/A	N/A	92.0	88.1	95.9
Western	N/A	N/A	N/A	N/A	N/A	N/A	N/A	N/A	N/A	79.3	71.3	87.3
PCV vaccine (12-23m)	1999			2004			2010			2015		
	%	95% CI		%	95% CI		%	95% CI		%	95% CI	
Mainland	N/A	N/A	N/A	N/A	N/A	N/A	N/A	N/A	N/A	86.8	84.3	89.3
Central	N/A	N/A	N/A	N/A	N/A	N/A	N/A	N/A	N/A	96.2	92.1	100.3
Eastern	N/A	N/A	N/A	N/A	N/A	N/A	N/A	N/A	N/A	92.6	89.3	95.9
Lake	N/A	N/A	N/A	N/A	N/A	N/A	N/A	N/A	N/A	83.2	78.3	88.1
Northern	N/A	N/A	N/A	N/A	N/A	N/A	N/A	N/A	N/A	95.5	92.6	98.4
Southern	N/A	N/A	N/A	N/A	N/A	N/A	N/A	N/A	N/A	93.2	88.7	97.7
Southern Highlands	N/A	N/A	N/A	N/A	N/A	N/A	N/A	N/A	N/A	88.4	83.3	93.5
Western	N/A	N/A	N/A	N/A	N/A	N/A	N/A	N/A	N/A	74.9	66.1	83.7
DPT3 vaccine (12-23m)	1999			2004			2010			2015		
	%	95% CI		%	95% CI		%	95% CI		%	95% CI	

Mainland	80.9	74.2	87.6	85.9	82.4	89.4	87.9	85.2	90.6	89.6	87.2	92.0
Central	79.6	62.2	97.0	91.9	81.7	102.1	95.3	91.2	99.4	97.3	94.8	99.8
Eastern	89.9	72.7	107.1	93.7	88.6	98.8	98.0	94.7	101.3	94.0	90.9	97.1
Lake	78.9	70.9	86.9	89.6	83.1	96.1	91.4	87.5	95.3	89.5	85.4	93.6
Northern	82.0	59.7	104.3	86.6	79.0	94.2	91.7	86.8	96.6	96.0	93.3	98.7
Southern	94.7	87.4	102.0	96.5	93.6	99.4	94.0	89.3	98.7	93.2	88.7	97.7
Southern Highlands	87.8	77.0	98.6	84.6	75.8	93.4	82.1	72.3	91.9	90.2	85.9	94.5
Western	63.8	45.4	82.2	73.6	64.8	82.4	74.9	67.8	82.0	79.0	70.4	87.6
Measles vaccine (12-23m)	1999			2004			2010			2015		
	%	95% CI		%	95% CI		%	95% CI		%	95% CI	
Mainland	78.9	73.2	84.6	80.0	76.3	83.7	84.6	82.1	87.1	85.9	83.5	88.3
Central	79.4	61.2	97.6	86.4	76.0	96.8	88.2	82.5	93.9	93.3	89.0	97.6
Eastern	86.2	79.1	93.3	87.8	81.3	94.3	95.2	91.1	99.3	91.8	88.5	95.1
Lake	77.5	70.6	84.4	84.8	78.5	91.1	89.5	84.8	94.2	86.6	82.3	90.9
Northern	90.5	79.3	101.7	85.7	79.2	92.2	88.1	82.2	94.0	89.5	84.2	94.8
Southern	94.9	89.2	100.6	91.9	87.6	96.2	90.1	84.8	95.4	89.1	83.6	94.6
Southern Highlands	81.7	70.1	93.3	77.9	65.6	90.2	84.2	77.5	90.9	85.2	78.7	91.7
**Treatment of childhood illness**												
ORS for diarrhea	1999			2004			2010			2015		
	%	95% CI		%	95% CI		%	95% CI		%	95% CI	
Mainland	55.7	47.9	63.5	54.7	50.2	59.2	45.2	40.9	49.5	44.6	40.7	48.5
Central	55.4	16.6	94.2	61.2	51.4	71.0	43.8	32.8	54.8	41.4	26.1	56.7
Eastern	33.8	18.7	48.9	70.4	54.9	85.9	46.1	33.0	59.2	41.3	30.1	52.5
Lake	48.4	41.9	54.9	60.1	48.5	71.7	40.7	32.1	49.3	45.1	36.7	53.5
Northern	60.2	37.3	83.1	44.0	32.2	55.8	51.0	38.1	63.9	39.0	26.5	51.5
Southern	70.0	54.5	85.5	60.0	50.0	70.0	42.3	30.9	53.7	50.4	39.2	61.6
Southern Highlands	55.9	37.3	74.5	54.8	44.4	65.2	47.0	36.0	58.0	44.6	37.7	51.5
Western	67.1	48.9	85.3	46.4	37.0	55.8	49.0	37.0	61.0	47.9	38.5	57.3
Zinc for diarrhea	1999			2004			2010			2015		

	%	95% CI		%	95% CI		%	95% CI		%	95% CI	
Mainland	N/A	N/A	N/A	N/A	N/A	N/A	5.1	3.1	7.1	17.2	14.5	19.9
Central	N/A	N/A	N/A	N/A	N/A	N/A	4.9	0.0	9.8	14.5	4.1	24.9
Eastern	N/A	N/A	N/A	N/A	N/A	N/A	8.4	0.4	16.4	17.1	9.3	24.9
Lake	N/A	N/A	N/A	N/A	N/A	N/A	0.3	-0.3	0.9	18.9	13.2	24.6
Northern	N/A	N/A	N/A	N/A	N/A	N/A	2.8	-1.5	7.1	19.3	10.1	28.5
Southern	N/A	N/A	N/A	N/A	N/A	N/A	10.9	2.5	19.3	13.5	6.1	20.9
Southern Highlands	N/A	N/A	N/A	N/A	N/A	N/A	10.8	3.7	17.9	14.2	7.7	20.7
Western	N/A	N/A	N/A	N/A	N/A	N/A	4.8	-0.9	10.5	18.7	12.2	25.2
Careseeking for pneumonia		1999			2004			2010			2015	
	%	95% CI		%	95% CI		%	95% CI		%	95% CI	
Mainland	81.1	72.9	89.3	59.0	53.3	64.7	62.9	57.2	68.6	50.4	44.9	55.9
Central	75.9	34.7	117.1	56.4	38.2	74.6	58.4	43.5	73.3	50.7	25.0	76.4
Eastern	91.2	79.2	103.2	63.3	48.0	78.6	82.9	67.6	98.2	71.4	59.6	83.2
Lake	73.0	62.8	83.2	68.2	55.1	81.3	51.8	40.8	62.8	48.3	37.9	58.7
Northern	80.5	50.1	110.9	70.5	57.6	83.4	80.0	66.7	93.3	64.7	51.2	78.2
Southern	84.1	73.7	94.5	73.2	59.7	86.7	84.8	71.7	97.9	73.9	54.3	93.5
Southern Highlands	88.9	83.4	94.4	58.2	44.3	72.1	45.9	29.0	62.8	34.4	19.9	48.9
Western	79.4	68.4	90.4	47.0	36.2	57.8	59.8	47.8	71.8	34.4	22.8	46.0
Any antimalarial drug		1999			2004			2010			2015	
	%	95% CI		%	95% CI		%	95% CI		%	95% CI	
Mainland	53.8	46.7	60.9	59.7	55.8	63.6	60.9	57.6	64.2	52.8	49.7	55.9
Central	61.7	41.5	81.9	49.8	40.0	59.6	54.0	44.6	63.4	25.8	14.2	37.4
Eastern	61.3	37.6	85.0	65.9	54.5	77.3	63.2	54.2	72.2	57.5	50.2	64.8
Lake	50.3	39.9	60.7	54.8	44.8	64.8	62.5	55.6	69.4	55.8	49.1	62.5
Northern	38.0	20.9	55.1	49.2	37.6	60.8	45.3	35.3	55.3	29.6	21.2	38.0
Southern	58.6	46.1	71.1	67.3	59.5	75.1	77.1	70.0	84.2	62.7	53.7	71.7
Southern Highlands	66.8	52.1	81.5	50.0	41.2	58.8	61.4	48.9	73.9	32.1	22.9	41.3
Western	63.5	53.5	73.5	65.9	58.5	73.3	64.0	57.3	70.7	64.2	58.5	69.9
ACT for Malaria		1999			2004			2010			2015	

	%	95% CI		%	95% CI		%	95% CI		%	95% CI	
Mainland	N/A	N/A	N/A	21.5	18.8	24.2	38.4	34.9	41.9	44.9	41.6	48.2
Central	N/A	N/A	N/A	14.4	6.6	22.2	41.0	32.8	49.2	21.4	10.8	32.0
Eastern	N/A	N/A	N/A	19.5	12.1	26.9	38.0	29.2	46.8	47.1	38.1	56.1
Lake	N/A	N/A	N/A	17.2	10.5	23.9	38.3	30.7	45.9	47.4	40.7	54.1
Northern	N/A	N/A	N/A	15.8	8.7	22.9	30.6	21.6	39.6	26.8	18.6	35.0
Southern	N/A	N/A	N/A	25.8	19.5	32.1	55.4	45.0	65.8	60.5	51.5	69.5
Southern Highlands	N/A	N/A	N/A	19.9	12.3	27.5	36.6	25.2	48.0	28.5	19.9	37.1
Western	N/A	N/A	N/A	27.3	21.8	32.8	36.3	28.7	43.9	52.3	45.6	59.0
ITN treated in previous 6 months	1999			2004			2010			2015		
	%	95% CI		%	95% CI		%	95% CI		%	95% CI	
Mainland	8.5	5.0	12.0	23.1	20.6	25.6	63.4	61.6	65.2	65.4	63.6	67.2
Central	29.3	4.8	53.8	13.7	8.0	19.4	59.8	53.5	66.1	41.0	34.1	47.9
Eastern	17.0	9.2	24.8	49.4	41.8	57.0	54.8	50.1	59.5	62.5	57.6	67.4
Lake	1.0	-0.6	2.6	23.5	17.8	29.2	75.9	72.2	79.6	91.3	88.9	93.7
Northern	4.7	-3.7	13.1	15.4	10.5	20.3	56.0	50.9	61.1	46.1	41.6	50.6
Southern	4.8	-5.2	14.8	20.4	14.9	25.9	66.1	63.0	69.2	65.2	61.7	68.7
Southern Highlands	6.1	-1.0	13.2	13.1	7.6	18.6	57.7	53.4	62.0	48.4	43.7	53.1
Western	9.6	-0.4	19.6	21.5	17.2	25.8	73.5	70.0	77.0	90.4	87.9	92.9
**Water Sanitation & Hygiene (WASH)**												
Improved drinking water source	1999			2004			2010			2015		
	%	95% CI		%	95% CI		%	95% CI		%	95% CI	
Mainland	72.1	67.6	76.6	51.6	47.1	56.1	56.2	52.9	59.5	66.2	63.3	69.1
Central	49.3	29.3	69.3	66.4	56.0	76.8	50.7	39.9	61.5	62.2	49.1	75.3
Eastern	71.0	61.4	80.6	66.8	57.4	76.2	70.8	63.9	77.7	65.9	57.5	74.3
Lake	61.8	54.9	68.7	39.8	28.4	51.2	44.8	36.6	53.0	60.8	53.5	68.1
Northern	81.3	69.7	92.9	63.9	54.7	73.1	68.0	60.9	75.1	79.9	73.4	86.4
Southern	78.6	67.8	89.4	46.3	36.7	55.9	50.0	42.6	57.4	70.0	62.4	77.6
Southern Highlands	81.6	71.6	91.6	46.0	32.7	59.3	61.1	51.7	70.5	61.9	54.8	69.0

Western	69.3	54.8	83.8	42.0	30.6	53.4	42.6	32.4	52.8	62.2	55.5	68.9
Water connection in the household	1999			2004			2010			2015		
	%	95% CI		%	95% CI		%	95% CI		%	95% CI	
Mainland	15.1	10.6	19.6	6.4	4.6	8.2	7.4	5.6	9.2	10.6	8.8	12.4
Central	6.8	-3.0	16.6	2.7	0.0	5.4	1.5	-1.0	4.0	4.4	1.9	6.9
Eastern	38.3	24.0	52.6	12.2	5.7	18.7	7.9	4.6	11.2	11.5	7.0	16.0
Lake	9.5	2.6	16.4	4.6	0.5	8.7	5.1	1.2	9.0	5.4	2.5	8.3
Northern	16.8	4.6	29.0	13.1	8.0	18.2	13.3	8.0	18.6	23.6	18.1	29.1
Southern	4.0	1.6	6.4	2.7	0.2	5.2	6.3	2.0	10.6	6.1	3.0	9.2
Southern Highlands	13.2	3.8	22.6	4.0	0.7	7.3	10.2	4.3	16.1	10.8	5.1	16.5
Western	6.3	-2.9	15.5	3.8	0.9	6.7	4.6	1.3	7.9	7.9	4.8	11.0
Improved sanitary facility	1999			2004			2010			2015		
	%	95% CI		%	95% CI		%	95% CI		%	95% CI	
Mainland	57.6	52.3	62.9	63.2	60.7	65.7	56.9	54.5	59.3	59.4	57.4	61.4
Central	48.1	35.9	60.3	55.6	47.0	64.2	43.2	35.8	50.6	54.2	47.7	60.7
Eastern	51.1	38.9	63.3	56.9	50.0	63.8	51.9	45.6	58.2	51.3	45.0	57.6
Lake	62.1	49.9	74.3	61.1	55.0	67.2	55.0	49.9	60.1	59.2	54.5	63.9
Northern	45.5	30.8	60.2	61.4	54.7	68.1	49.0	43.5	54.5	57.8	52.7	62.9
Southern	76.9	69.1	84.7	84.9	81.8	88.0	74.3	69.2	79.4	73.0	69.1	76.9
Southern Highlands	69.8	62.5	77.1	74.0	66.6	81.4	67.4	60.7	74.1	65.1	60.2	70.0
Western	60.7	55.4	66.0	57.0	50.7	63.3	59.5	51.9	67.1	59.8	55.1	64.5
Hygienic disposal of stools	1999			2004			2010			2015		
	%	95% CI		%	95% CI		%	95% CI		%	95% CI	
Mainland	N/A	N/A	N/A	68.5	64.6	72.4	64.7	62.0	67.4	70.7	68.5	72.9
Central	N/A	N/A	N/A	72.0	62.2	81.8	56.6	48.2	65.0	71.2	62.4	80.0
Eastern	N/A	N/A	N/A	90.1	86.4	93.8	85.7	81.0	90.4	78.8	74.7	82.9
Lake	N/A	N/A	N/A	48.7	42.0	55.4	51.4	45.5	57.3	65.7	61.4	70.0
Northern	N/A	N/A	N/A	74.8	66.8	82.8	60.8	52.0	69.6	67.8	61.5	74.1
Southern	N/A	N/A	N/A	89.4	86.1	92.7	88.1	84.8	91.4	90.0	86.7	93.3
Southern Highlands	N/A	N/A	N/A	75.1	60.8	89.4	64.9	56.7	73.1	82.4	78.5	86.3

Western	N/A	N/A	N/A	61.5	53.3	69.7	63.5	57.0	70.0	60.6	55.7	65.5

Appendix S6. Breastfeeding Practices by Zone

	1999			2004			2010			2015		
	%	95% CI		%	95% CI		%	95% CI		%	95% CI	
Early initiation of breastfeeding												
Mainland	N/A	59.0	55.3	62.7	47.2	44.8	49.6	51.8	49.6	54.0	N/A	59.0
Central	N/A	67.5	60.2	74.8	40.4	32.8	48.0	69.0	63.9	74.1	N/A	67.5
Eastern	N/A	75.5	70.4	80.6	68.4	62.7	74.1	57.3	51.8	62.8	N/A	75.5
Lake	N/A	80.5	73.2	87.8	33	29.1	36.9	37.7	33.2	42.2	N/A	80.5
Northern	N/A	59.6	53.7	65.5	63.4	56.9	69.9	75.3	71.6	79.0	N/A	59.6
Southern	N/A	53.4	48.5	58.3	52.4	46.7	58.1	57.4	52.1	62.7	N/A	53.4
Southern Highlands	N/A	45.8	41.1	50.5	41.9	35.4	48.4	54.4	49.9	58.9	N/A	45.8
Western	N/A	36.2	30.3	42.1	42.7	37.0	48.4	37.5	32.6	42.4	N/A	36.2
Exclusive breastfeeding (0-5 months)												
Mainland	34.9	25.5	44.3	42.1	37.6	46.6	49.7	45.4	54.0	62.7	59.0	66.4
Central	24.3	8.4	40.2	45.0	36.8	53.2	59.5	48.3	70.7	75.6	64.6	86.6
Eastern	12.1	-1.0	25.2	27.4	15.4	39.4	26.1	14.3	37.9	46.2	34.8	57.6
Lake	53.3	36.8	69.8	61.5	52.3	70.7	62.7	55.4	70.0	63.1	55.8	70.4
Northern	39.3	17.5	61.1	21.8	12.8	30.8	48.9	35.4	62.4	53.9	44.3	63.5
Southern	26.4	7.0	45.8	22.8	12.4	33.2	44.6	33.2	56.0	40.2	20.0	60.4
Southern Highlands	31.7	13.9	49.5	41.5	29.2	53.8	43.5	28.8	58.2	72.3	62.5	82.1
Western	42.0	24.8	59.2	46.1	36.3	55.9	49.2	40.4	58.0	74.0	67.5	80.5
Predominant breastfeeding (0-5 months)												
Mainland	35.6	27.8	43.4	19.9	16.8	23.0	9.7	7.0	12.4	12.5	10.0	15.0
Central	46.5	19.5	73.5	3.7	-0.6	8.0	11.4	3.0	19.8	6.7	0.4	13.0
Eastern	45.6	28.4	62.8	22.5	13.1	31.9	15.6	4.0	27.2	20.5	8.0	33.0
Lake	17.4	6.8	28.0	17.0	9.6	24.4	6.7	0.8	12.6	12.4	8.3	16.5
Northern	29.0	12.7	45.3	23.4	15.0	31.8	8.5	1.8	15.2	13.7	7.6	19.8
Southern	54.5	32.0	77.0	22.1	11.9	32.3	4.4	-0.9	9.7	31.1	17.8	44.4
Southern Highlands	48.6	28.2	69.0	13.9	6.5	21.3	8.1	2.0	14.2	7.1	1.6	12.6
Western	36.8	21.3	52.3	28.4	21.1	35.7	12.2	6.3	18.1	7.1	3.8	10.4
Partial breastfeeding (0-5 months)												
Mainland	26.9	20.4	33.4	35.8	31.5	40.1	38.8	34.3	43.3	23.0	19.5	26.5
Central	29.2	5.3	53.1	45.3	36.1	54.5	29.1	19.9	38.3	17.6	7.6	27.6
Eastern	42.3	27.6	57.0	50.2	38.0	62.4	55.7	38.5	72.9	31.4	20.0	42.8
Lake	20.9	9.3	32.5	21.0	13.0	29.0	26.8	20.5	33.1	21.4	12.8	30.0
Northern	31.7	18.0	45.4	53.8	41.8	65.8	42.6	28.9	56.3	29.8	20.8	38.8
Southern	19.1	-4.8	43.0	55.1	42.6	67.6	51.0	40.4	61.6	28.7	14.0	43.4
Southern Highlands	19.7	4.0	35.4	41.6	29.3	53.9	48.4	32.3	64.5	19.6	10.2	29.0
Western	15.9	2.8	29.0	21.6	14.2	29.0	35.9	27.3	44.5	17.7	12.0	23.4
Any breastfeeding (6-11 months)												
Mainland	96.5	93.6	99.4	97.4	95.6	99.2	96.4	94.6	98.2	98.1	96.9	99.3
Central	100.0	100.0	100.0	92.7	86.0	99.4	99.2	97.6	100.8	100.0	100.0	100.0

Eastern	100.0	100.0	100.0	98.9	96.7	101.1	87.4	75.2	99.6	94.2	87.9	100.5
Lake	98.2	94.7	101.7	99.3	97.9	100.7	96.7	93.8	99.6	99.3	97.9	100.7
Northern	98.9	96.5	101.3	98.6	95.9	101.3	100.0	100.0	100.0	97.9	95.0	100.8
Southern	100.0	100.0	100.0	100.0	100.0	100.0	98.7	96.2	101.2	97.6	93.1	102.1
Southern Highlands	98.8	96.4	101.2	95.4	88.0	102.8	98.0	95.3	100.7	99.6	98.8	100.4
Western	83.4	68.7	98.1	96.8	93.3	100.3	95.7	92.2	99.2	97.6	95.4	99.8
Any breastfeeding (12-23 months)												
Mainland	78.9	74.0	83.8	77.5	74.6	80.4	76.6	73.7	79.5	75.4	73.2	77.6
Central	85.6	72.7	98.5	83.3	75.7	90.9	80.1	71.1	89.1	84.2	75.6	92.8
Eastern	78.7	70.1	87.3	73.4	65.0	81.8	80.0	72.6	87.4	77.1	71.2	83.0
Lake	76.3	69.0	83.6	68.5	61.2	75.8	69.1	61.5	76.7	67.9	62.2	73.6
Northern	69.2	51.2	87.2	82.2	76.3	88.1	83.2	77.7	88.7	82.4	77.3	87.5
Southern	90.9	83.1	98.7	97.0	94.1	99.9	88.8	82.1	95.5	80.9	74.4	87.4
Southern Highlands	90.6	83.2	98.0	83.4	77.7	89.1	77.3	69.7	84.9	72.3	65.8	78.8
Western	75.3	63.5	87.1	72.7	67.0	78.4	72.0	66.1	77.9	74.0	69.7	78.3

Appendix S7. Under-five mortality estimated by the Lives Saved Tool and Demographic Health Surveys

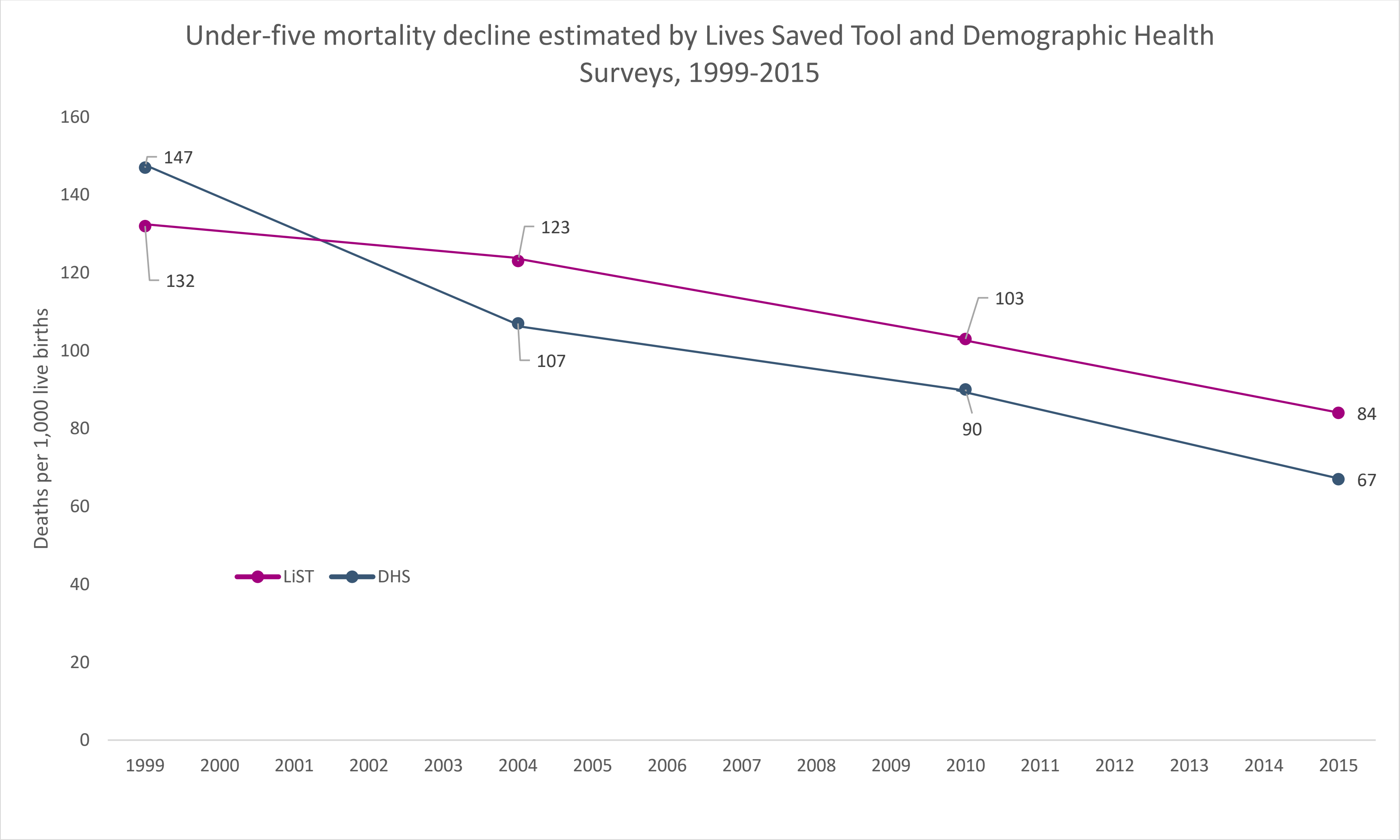

Supplement: Online Supplementary Document [file jogh-08-021201-s001.pdf]
